# 多维生活方式和社会经济地位与肺癌发病的关联及交互作用

**DOI:** 10.3779/j.issn.1009-3419.2025.106.19

**Published:** 2025-07-20

**Authors:** Haotian LIU, Runhuang YANG, Haiping ZHANG, Shiyun LV, Bo GAO, Lixin TAO, Yanxia LUO, Xiuhua GUO

**Affiliations:** ^1^100069 北京，首都医科大学公共卫生学院; ^1^School of Public Health, Capital Medical University, Beijing 100069, China; ^2^100069 北京，环境与衰老北京市重点实验室; ^2^Beijing Key Laboratory of Environment and Aging, Beijing 100069, China; ^3^100069 北京，首都医科大学健康医疗大数据国家研究院; ^3^National Institute for Data Science in Health and Medicine, Capital Medical University, Beijing 100069, China

**Keywords:** 肺肿瘤, 多维生活方式, 社会经济地位, *Cox*比例风险模型, Lung neoplasms, Multidimensional lifestyle, Socioeconomic status, *Cox* proportional hazards model

## Abstract

**背景与目的** 肺癌发病率和死亡率持续攀升，高危人群筛查与早期预防的需求十分迫切。本研究旨在探索多维度生活方式和社会经济地位与肺癌发病的关联及其交互作用，为肺癌高危人群筛查和预防提供科学依据。**方法** 通过问卷调查获得的吸烟、饮酒、锻炼、饮食和睡眠信息构建健康生活方式评分，根据教育、就业和家庭收入信息对社会经济地位进行评估，并使用基因检测数据评估遗传变异风险。进行等比例风险假设检验，应用*Cox*比例风险模型，分析调整遗传变异风险、年龄、性别、糖尿病、高血压和生活环境评分后，健康生活方式评分和社会经济地位与肺癌的关联，以及各因素之间的交互作用。**结果** 共纳入2006年3月至2010年10月进入队列的样本245,538例，并随访至2022年12月31日，将参与者分为病例组（*n*=1472）和对照组（*n*=244,066）。分析结果显示，在调整协变量后，健康生活方式评分和社会经济地位与肺癌发病之间仍存在关联：与高健康生活方式评分的参与者相比，中、低健康生活方式评分的参与者患肺癌的风险显著提高，风险比（hazard ratio, HR）分别为2.12（95%CI: 1.86-2.41）和3.36（95%CI: 2.82-3.99）；与高社会经济地位的参与者相比，中、低社会经济地位的参与者患肺癌的风险显著提高，HR分别为1.29（95%CI: 1.13-1.48）和1.67（95%CI: 1.46-1.90）。是否吸烟与社会经济地位（*P*_for interaction_=0.05）、其他4种生活方式（*P*_for interaction_=0.02）之间存在交互作用。**结论** 本研究发现了多维生活方式及社会经济地位与肺癌发病之间存在关联，以及吸烟与社会经济地位、其他4种生活方式之间的交互作用，为肺癌高危人群筛查和预防提供了科学依据。

## Abstract (en)

**Background and objective** The incidence and mortality rates of lung cancer remain on the rise, creating an urgent need for screening among high-risk populations and early prevention. This study aims to explore the association and interaction between multidimensional lifestyle, socioeconomic status, and the incidence of lung cancer, and to provide scientific evidence for screening high-risk populations and preventing lung cancer. **Methods** Healthy lifestyle score was constructed using information on smoking, alcohol consumption, exercise, diet and sleep obtained through a questionnaire survey. Socioeconomic status was evaluated based on information on education, employment, and family income, and genetic testing data were used to assess the risk of genetic variation. A proportional hazards assumption test was conducted, and the *Cox* proportional hazards model was applied to analyze the associations between healthy lifestyle scores, socioeconomic status, and lung cancer, as well as the interactions among various factors, after adjusting for the risk of genetic variation, age, gender, diabetes, hypertension and the living environment score. **Results** A total of 245,538 samples that entered the cohort from March, 2006 to October, 2010 were included and followed up until December 31, 2022. The participants were divided into the case group (*n*=1472) and the control group (*n*=244,066). The analysis results showed that after adjusting for covariates, there was still an association between the healthy lifestyle score, socioeconomic status, and the incidence of lung cancer: compared with participants with a high healthy lifestyle score, the risk of lung cancer in participants with medium and low healthy lifestyle scores was significantly increased, with hazard ratios (HR) of 2.12 (95%CI: 1.86-2.41) and 3.36 (95%CI: 2.82-3.99) respectively; compared with participants with high socioeconomic status, the risk of lung cancer in participants with medium and low socioeconomic status was significantly increased, with HR of 1.29 (95%CI: 1.13-1.48) and 1.67 (95%CI: 1.46-1.90) respectively. Moreover, there were interactions between smoking status and socioeconomic status (*P*_for interaction_=0.05), as well as the other four lifestyle factors (*P*_for interaction_=0.02). **Conclusion** This study identified the association between multidimensional lifestyle factors and socioeconomic status with the incidence of lung cancer, as well as interactions between smoking and socioeconomic status and four other lifestyle factors, providing a scientific basis for screening and prevention in high-risk populations for lung cancer.

近年来肺癌发病率和死亡率持续攀升，2022年全球新发肺癌病例超过248万，死亡病例超过180万，重新成为发病率与致死率最高的恶性肿瘤^[1,2]^。肺癌的5年生存率很低，具体取决于诊断时的疾病阶段^[3,4]^，但超过75%的肺癌患者被诊断为晚期肺癌，这显示了肺癌高危人群筛查与早期预防的迫切需求。因此，识别肺癌的危险因素，并对危险因素进行提前干预，对肺癌的防治工作尤为重要。

肺癌的机制十分复杂，涉及生活方式、社会和遗传等多维因素。吸烟被公认为是最重要的肺癌危险因素^[5,6]^，且近年来许多研究逐渐揭示饮食、睡眠及运动等生活方式以及文化水平、家庭收入等社会决定因素在肺癌发病中的协同作用^[7,8]^，而根据单核苷酸多态性（single nucleotide polymorphism, SNP）评估的遗传变异风险也被证明可能与肺癌发病及预后存在相关性^[9,10]^。然而，目前大部分研究缺乏对这些因素的综合评价，只是对单种或几种因素与肺癌发生之间的关联进行分析；并且在排除掉遗传变异风险的影响后，这种综合评价与肺癌之间是否存在关联，也尚不清楚。

本研究旨在根据多维生活方式和社会决定因素构建健康生活方式评分和评估社会经济地位，探索它们与肺癌发病之间的关联，以及调整遗传变异风险的影响后关联是否仍然存在，并对不同生活方式之间以及它们和社会经济地位之间的交互作用进行分析，为肺癌高危人群筛查和预防提供科学依据。

## 1 资料与方法

### 1.1 研究对象

英国生物银行（UK Biobank, UKB）为一项前瞻性队列研究，在2006年3月至2010年10月招募志愿者进入队列并进行随访。本研究剔除在基线时患有癌症（*n*=46,624）、问卷信息缺失（*n*=176,345）、无基因检测和其他协变量缺失（*n*=33,862）的参与者，最终纳入样本245,538例，筛选流程见图1。参与者完成了关于饮食、生活方式和健康相关信息的问卷以及身体测量，并随访至2022年12月31日，中位随访时间为13.82年。在随访期间患肺癌的参与者设为病例组，随访结束时未患肺癌的参与者设为对照组。本研究的数据使用通过了UKB的数据申请（申请号为88589）。

**图1:**
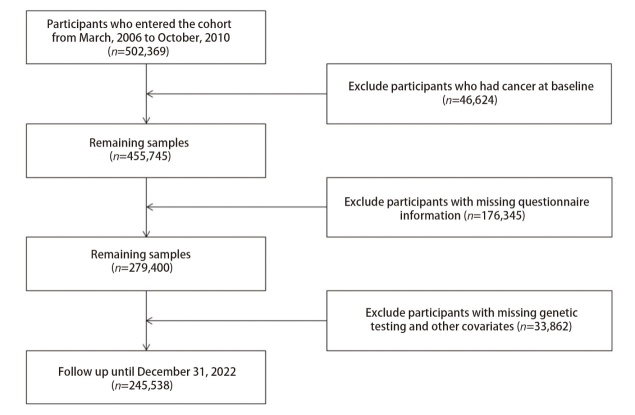
研究对象筛选流程图

### 1.2 研究资料与方法

#### 1.2.1 健康生活方式评分

本研究使用5种生活方式因素，即是否吸烟、饮酒水平、健康睡眠、健康饮食和充足锻炼，建立了健康生活方式评分。是否吸烟根据目前是否吸烟和过去是否吸烟判断，目前吸烟或过去吸烟满足一项为是，目前或过去均不吸烟为否；饮酒水平根据饮酒频率判断，每月饮酒超过3次为不健康水平，每月饮酒不超过3次为健康水平；充足锻炼根据代谢当量（metabolic equivalent of task, MET）是否达到中度或剧烈活动标准值判断，达到标准为是，未达到标准为否。健康饮食通过基线获得的食物频率问卷进行评估，根据蔬菜、水果、加工肉类、红肉、鱼肉、全谷物和精制谷物的摄入量计算饮食质量评分^[11]^，每满足以下一项标准则增加1分：（1）蔬菜≥每天3份；（2）水果≥每天3份；（3）加工肉类≤每周1份；（4）红肉≤每周1.5份；（5）鱼肉≥每周2份；（6）全谷物≥每周3份；（7）精制谷物≤每周1.5份，最高为7分，≥4分为是，<4分为否。健康睡眠根据睡眠时间类型、睡眠持续时间、失眠频率、打鼾和白天是否嗜睡计算睡眠质量评分进行评估^[12]^，每满足以下一项标准则增加1分：（1）早睡型睡眠时间；（2）每天睡眠7-8 h；（3）从未或很少出现失眠症状；（4）无自我报告的打鼾；（5）无频繁的白天嗜睡，最高为5分，≥3分为是，<3分为否。每满足一个健康的生活方式（从不吸烟、每月饮酒不超过3次、MET达到标准值、饮食质量评分≥4分、睡眠质量评分≥3分），健康生活方式评分增加1分，最高为5分。0-1分为低健康生活方式评分，2-3分为中健康生活方式评分，4-5分为高健康生活方式评分。

#### 1.2.2 社会经济地位

本研究根据文化水平（高中以下、高中及以上）、就业情况（在职、退休、失业）和税前家庭总收入（低于31,000英镑为低水平，不低于31,000英镑为高水平）3个因素，对参与者的社会经济地位进行判断^[13]^。UKB数据库对文化水平、就业情况和收入情况进行了评分，并生成了教育评分、就业评分和收入评分3个指标，将3个指标求和，并根据求和的三分位数将参与者分为低、中或高社会经济地位。

#### 1.2.3 遗传变异风险

对SNP质量控制后进行关联性分析，并筛选*P*<10^-4^的SNP位点125个，根据风险等位基因的数量将每个SNP的效应量编码为0、1和2，并通过相应的比值比（odds ratio, OR）值进行加权。计算每位参与者的加权遗传风险评分，并根据评分的三分位数将参与者分为肺癌的低、中或高遗传变异风险^[14]^。

#### 1.2.4 其他协变量

包括年龄（60岁以下、60岁及以上）、性别（男、女）、糖尿病（是、否）、高血压（是、否）、体质指数（body mass index, BMI）（低、中、高），生活环境评分（低、中、高）。

#### 1.2.5 结局事件定义

肺癌诊断依据UKB数据库中的癌症登记。诊断标准基于国际疾病分类（international classification of diseases, ICD）代码，包括ICD-10中的C34.1、C34.2、C34.3、C34.8和C34.9。

### 1.3 统计分析

统计分析软件采用R 4.4.1。使用χ^2^检验比较基线特征的组间差异（*P*<0.05）。采用*Schoenfeld*残差检验进行等比例风险假设检验（*P*>0.05）。以结局事件发生时间作为时间尺度，应用*Cox*比例风险模型分析健康生活方式评分和社会经济地位与肺癌发病之间的关联，将年龄、性别、是否患糖尿病、是否患高血压、BMI和生活环境评分中满足等比例风险假设检验的变量作为协变量对模型进行调整，得到各因素的风险比（hazard ratio, HR）及其95%置信区间（confidence interval, CI），并绘制森林图实现结果可视化。为证明健康生活方式评分选取的生活方式的合理性，对各生活方式与肺癌发病的关联性进行分析，并对它们的风险大小进行比较。为进一步探索不同因素之间是否存在相互影响，对不同生活方式和社会经济地位在肺癌发生中的交互作用进行评估，获得HR及其95%CI和*P*值。*P*<0.05为差异具有统计学意义。

## 2 结果

### 2.1 问卷信息统计描述和单因素分析

本研究共纳入2006年3月至2010年10月进入队列的样本245,538例，随访至2022年12月31日（中位随访时间为13.82年），将样本分为病例组（1472例）和对照组（244,066例），发病率为0.60%。健康生活方式评分及5种生活方式统计描述和单因素分析见表1，社会经济地位及相关变量的统计描述和单因素分析见表2，其他协变量的统计描述和单因素分析见表3。通过χ^2^检验发现，BMI在病例组和对照组之间不存在统计学差异（*P*=0.176），其余变量在病例组和对照组之间均存在统计学差异（*P*<0.05）。

**表1:** 健康生活方式评分及5种生活方式统计描述和单因素分析

Characteristics	Total (n=245,538)	Incidence rate	Control group (n=244,066)	Case group (n=1472)	χ^2^	P
Healthy lifestyle score					257.7	<0.001
Low	19,299 (7.9%)	1.17%	19,073 (7.8%)	226 (15.3%)		
Moderate	130,011 (52.9%)	0.72%	129,078 (52.9%)	933 (63.4%)		
High	96,228 (39.2%)	0.33%	95,915 (39.3%)	313 (21.3%)		
Smoke					481.7	<0.001
Yes	146,385 (59.6%)	0.88%	145,095 (59.4%)	1290 (87.6%)		
No	99,153 (40.4%)	0.18%	98,971 (40.6%)	182 (12.4%)		
Drinking level					65.6	<0.001
Healthy	189,427 (77.1%)	0.53%	188,422 (77.2%)	1005 (68.3%)		
Unhealthy	56,111 (22.9%)	0.83%	55,644 (22.8%)	467 (31.7%)		
Healthy diet					7.9	0.005
Yes	167,544 (68.2%)	0.57%	166,590 (68.3%)	954 (64.8%)		
No	77,994 (31.8%)	0.66%	77,476 (31.7%)	518 (35.2%)		
Healthy sleep					33.0	<0.001
Yes	179,635 (73.2%)	0.54%	178,656 (73.2%)	979 (66.5%)		
No	65,903 (26.8%)	0.75%	65,410 (26.8%)	493 (33.5%)		
Adequate exercise					6.2	0.013
Yes	132,106 (53.8%)	0.56%	131,362 (53.8%)	744 (50.5%)		
No	113,432 (46.2%)	0.64%	112,704 (46.2%)	728 (49.5%)		

**表2:** 社会经济地位及相关变量的统计描述和单因素分析

Characteristics	Total (n=245,538)	Incidence rate	Control group (n=244,066)	Case group (n=1472)	χ^2^	P
Socioeconomic status					55.2	<0.001
Low	81,870 (33.3%)	0.75%	81,258 (33.3%)	612 (41.6%)		
Moderate	81,912 (33.4%)	0.59%	81,432 (33.4%)	480 (32.6%)		
High	81,756 (33.3%)	0.46%	81,376 (33.3%)	380 (25.8%)		
Education					23.2	<0.001
High school or above	169,433 (69.0%)	0.55%	168,503 (69.0%)	930 (63.2%)		
Below high school	76,105 (31.0%)	0.71%	75,563 (31.0%)	542 (36.8%)		
Employment status					316.6	<0.001
Employed	163,851 (66.7%)	0.41%	163,184 (66.9%)	667 (45.3%)		
Retired	65,386 (26.6%)	1.03%	64,713 (26.5%)	673 (45.7%)		
Unemployed	16,301 (6.6%)	0.81%	16,169 (6.6%)	132 (9.0%)		
Income					295.7	<0.001
High	147,556 (60.1%)	0.38%	146,994 (60.2%)	562 (38.2%)		
Low	97,982 (39.9%)	0.93%	97,072 (39.8%)	910 (61.8%)		

**表3:** 协变量统计描述和单因素分析

Characteristics	Total (n=245,538)	Incidence rate	Control group (n=244,066)	Case group (n=1472)	χ^2^	P
Age (yr)					557.7	<0.001
≥60	86,138 (35.1%)	1.10%	85,190 (34.9%)	948 (64.4%)		
<60	159,400 (64.9%)	0.33%	158,876 (65.1%)	524 (35.6%)		
Gender					24.7	<0.001
Male	122,500 (49.9%)	0.68%	121,670 (49.9%)	830 (56.4%)		
Female	123,038 (50.1%)	0.52%	122,396 (50.1%)	642 (43.6%)		
Genetic variation risk					37.2	<0.001
Low	81,845 (33.3%)	0.49%	81,445 (33.4%)	400 (27.2%)		
Moderate	81,847 (33.3%)	0.59%	81,365 (33.3%)	482 (32.7%)		
High	81,846 (33.3%)	0.72%	81,256 (33.3%)	590 (40.1%)		
Hypertension					38.6	<0.001
Yes	53,440 (21.8%)	0.78%	53,021 (21.7%)	419 (28.5%)		
No	192,098 (78.2%)	0.55%	191,045 (78.3%)	1053 (71.5%)		
Diabetes					62.8	<0.001
Yes	10,751 (4.4%)	1.19%	10,624 (4.4%)	127 (8.6%)		
No	234,787 (95.6%)	0.57%	233,442 (95.6%)	1345 (91.4%)		
BMI					3.5	0.176
Low	1189 (0.5%)	0.93%	1178 (0.5%)	11 (0.7%)		
Moderate	81,771 (33.3%)	0.57%	81,303 (33.3%)	468 (31.8%)		
High	162,578 (66.2%)	0.61%	161,585 (66.2%)	993 (67.5%)		
Living environment score					19.9	<0.001
Low	81,857 (33.3%)	0.69%	81,294 (33.3%)	563 (38.2%)		
Moderate	81,898 (33.4%)	0.60%	81,407 (33.4%)	491 (33.4%)		
High	81,783 (33.3%)	0.51%	81,365 (33.3%)	418 (28.4%)		

BMI: body mass index.

### 2.2 健康生活方式评分和社会经济地位与肺癌发病的关联性分析

*Schoenfeld*残差检验结果显示，除BMI（*P*=0.037）外，其他变量的*P*值均>0.05，符合等比例风险假设。调整遗传变异风险、年龄、性别、糖尿病、高血压和生活环境评分后，使用*Cox*比例风险模型对健康生活方式评分和社会经济地位与肺癌发病的关联进行分析，并绘制森林图（图2）。结果显示，与高健康生活方式评分的参与者相比，中、低健康生活方式评分的参与者患肺癌的风险显著提高，HR分别为2.12（95%CI: 1.86-2.41）和3.36（95%CI: 2.82-3.99），中、低健康生活方式评分肺癌患者占全部肺癌患者的78.7%。与高社会经济地位的参与者相比，中、低社会经济地位的参与者患肺癌的风险显著提高，HR分别为1.29（95%CI: 1.13-1.48）和1.67（95%CI: 1.46-1.90），中、低社会经济地位肺癌患者占全部肺癌患者的74.2%。

**图2:**
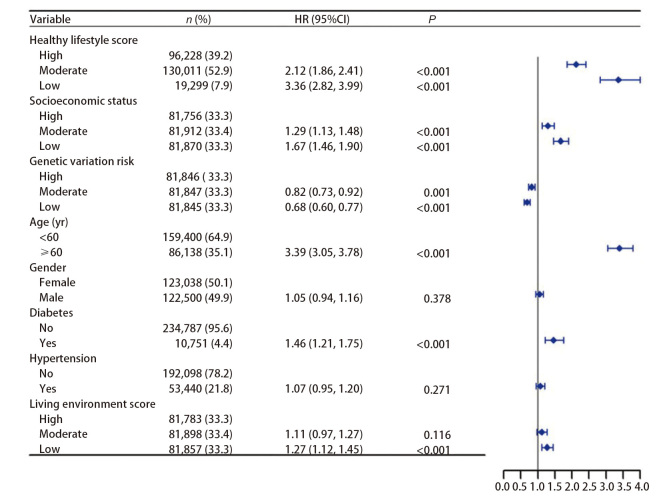
健康生活方式评分和社会经济地位与肺癌发病的关联性分析

### 2.3 各生活方式与肺癌发病的关联性分析及风险对比

*Schoenfeld*残差检验结果显示，除BMI（*P*=0.037）外，其他变量的*P*值均>0.05，符合等比例风险假设。模型结果（图3）显示，5种生活方式均会影响肺癌发病的风险（*P*<0.05）。通过比较HR值可以发现，吸烟对肺癌发生风险的影响最大（HR=4.24, 95%CI: 3.63-4.96），其他4种生活方式降序排列为：不健康睡眠（HR=1.23, 95%CI: 1.10-1.37）、不健康饮食（HR=1.21, 95%CI: 1.09-1.36）、不健康饮酒水平（HR=1.17, 95%CI: 1.05-1.31）、锻炼不充足（HR=1.15, 95%CI: 1.03-1.27）。

**图3:**
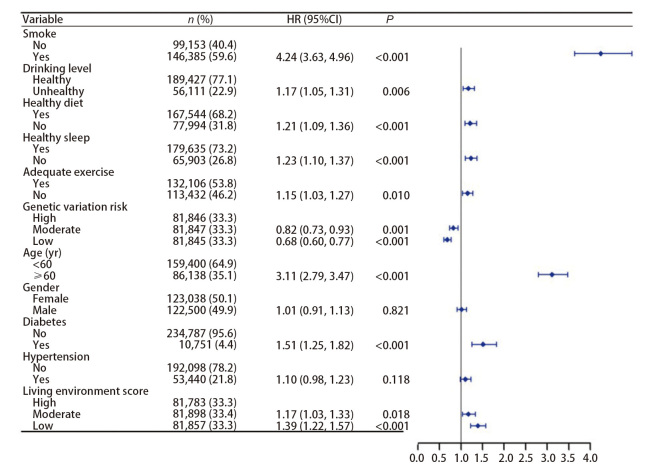
5种生活方式与肺癌发病的关联性分析

### 2.4 生活方式与社会经济地位的交互作用

对健康生活方式评分与社会经济地位做交互作用分析，结果（表4）显示两者没有明显的交互作用（*P*_for interaction_=0.585），但是在对各种生活方式与社会经济地位的交互作用分析结果显示，是否吸烟与社会经济地位之间存在相乘交互作用（*P*_for interaction_=0.050）。

**表4:** 生活方式与社会经济地位的交互作用分析（n=245,538）

Characteristics	n (%)	Socioeconomic status	HR (95%CI)	P	P for interaction
Healthy lifestyle score					0.585
High	96,228 (39.2)	High	Reference		
		Moderate	1.14 (0.85-1.52)	0.370	
		Low	1.58 (1.20-2.07)	0.001	
Moderate	130,011 (52.9)	High	Reference		
		Moderate	1.27 (1.07-1.50)	0.005	
		Low	1.51 (1.29-1.77)	<0.001	
Low	19,299 (7.9)	High	Reference		
		Moderate	1.42 (0.99-2.04)	0.056	
		Low	1.96 (1.40-2.75)	<0.001	
Smoke					0.050
No	99,153 (40.4)	High	Reference		
		Moderate	0.89 (0.62-1.28)	0.517	
		Low	1.09 (0.77-1.54)	0.630	
Yes	146,385 (59.6)	High	Reference		
		Moderate	1.33 (1.15-1.54)	<0.001	
		Low	1.68 (1.46-1.92)	<0.001	
Drinking level					0.813
Healthy	189,427 (77.1)	High	Reference		
		Moderate	1.28 (1.08-1.51)	0.005	
		Low	1.72 (1.47-2.01)	<0.001	
Unhealthy	56,111 (22.9)	High	Reference		
		Moderate	1.33 (1.07-1.67)	0.011	
		Low	1.65 (1.32-2.06)	<0.001	
Healthy diet					0.106
Yes	167,544 (68.2)	High	Reference		
		Moderate	1.27 (1.08-1.49)	0.004	
		Low	1.48 (1.26-1.73)	<0.001	
No	77,994 (31.8)	High	Reference		
		Moderate	1.24 (0.97-1.58)	0.081	
		Low	1.84 (1.48-2.29)	<0.001	
Healthy sleep					0.772
Yes	179,635 (73.2)	High	Reference		
		Moderate	1.26 (1.07-1.48)	0.005	
		Low	1.63 (1.39-1.90)	<0.001	
No	65,903 (26.8)	High	Reference		
		Moderate	1.23 (0.97-1.56)	0.094	
		Low	1.48 (1.18-1.86)	0.001	
Adequate exercise					0.658
Yes	132,106 (53.8)	High	Reference		
		Moderate	1.34 (1.11-1.62)	0.002	
		Low	1.66 (1.39-2.00)	<0.001	
No	113,432 (46.2)	High	Reference		
		Moderate	1.18 (0.98-1.43)	0.086	
		Low	1.55 (1.30-1.86)	<0.001	

### 2.5 吸烟与其他生活方式的交互作用

为了对肺癌预防提出具体建议，进一步对吸烟与其他4种生活方式进行了交互作用分析（表5），虽然吸烟和其他生活方式没有单独的交互作用，但是如果将其他4种生活方式进行综合评价，形成一个4种健康生活方式评分，有3-4种健康生活方式为高，0-2种健康生活方式为低，则吸烟和4种健康生活方式评分之间存在相乘交互作用（*P*_for interaction_=0.02）。

**表5:** 吸烟与其他4种生活方式的交互作用分析（n=245,538）

Characteristics	n (%)	HR (95%CI)	P	P for interaction
Other 4 lifestyle patterns score				0.020
High	137,585 (56.0)	3.67 (3.04-4.42)	<0.001	
Low	107,953 (44.0)	6.29 (4.16-9.51)	<0.001	
Drinking level				0.144
Healthy	189,427 (77.1)	4.39 (3.69-5.21)	<0.001	
Unhealthy	56,111 (22.9)	5.97 (4.10-8.69)	<0.001	
Healthy diet				0.061
Yes	167,544 (68.2)	4.34 (3.61-5.21)	<0.001	
No	77,994 (31.8)	6.02 (4.51-8.04)	<0.001	
Healthy sleep				0.710
Yes	179,635 (73.2)	4.67 (3.88-5.61)	<0.001	
No	65,903 (26.8)	4.98 (3.72-6.67)	<0.001	
Adequate exercise				0.543
Yes	132,106 (53.8)	5.06 (4.05-6.32)	<0.001	
No	113,432 (46.2)	4.59 (3.70-5.70)	<0.001	

## 3 讨论

本研究通过问卷收集到的吸烟、饮酒、饮食、睡眠和运动信息构建了一种健康生活方式评分，并根据文化水平、家庭收入和就业情况3个因素对参与者的社会经济地位进行了评估。通过*Cox*比例风险模型分析结果显示，在调整了遗传变异风险等协变量的影响后，健康生活方式评分和社会经济地位与肺癌之间仍存在关联，并且是否吸烟与社会经济地位、4种健康生活方式评分之间存在交互作用。

健康生活方式和社会经济地位与肺癌之间的关联与既往研究相似，即吸烟、饮食等生活方式与肺癌的发生发展之间存在关联^[15,16]^，教育程度等社会因素也与肺癌发病密切相关^[17,18]^。但是这些研究没有将各种生活方式和社会决定因素进行整合，也缺乏对不同生活方式以及社会经济地位之间的交互作用的分析。而本研究将多维生活方式和社会决定因素进行综合评价，更为全面地揭示了生活方式和社会经济地位与肺癌之间的关联。并且，本研究的交互作用分析结果对于肺癌预防具有重要意义。吸烟与社会经济地位在影响肺癌发病方面存在交互作用，这可能是由于社会经济地位高的人群收入水平和教育水平更高，可以获得的医疗资源优于中、低社会经济地位的人群，从而能够有效降低吸烟对于肺癌发病的影响。而吸烟与其他4种健康生活方式评分之间存在交互作用这一结果提示，对于已经吸烟的人群，尽可能采用多种健康生活方式，如摄入充足且丰富的食物，避免熬夜，充足睡眠，减少饮酒量，积极进行体育锻炼，仍能够有效降低患肺癌的风险。

本研究在构建健康生活方式评分时，考虑到该指标的作用为综合反映受试者生活方式健康水平，5种生活方式的重要性相当，因此计算评分时并未对5种生活方式进行加权。但是这种非加权的评分方式可能会低估吸烟这一生活方式对肺癌的影响，从而影响结果的准确性和筛选肺癌高危人群的精准性。为降低此方式的负面作用，本研究在进行*Cox*回归分析时，进一步对5种生活方式各自的HR进行了计算，结果显示是否吸烟对肺癌发生风险的影响远高于其他4种生活方式，这也提示了在进行肺癌高危人群筛选时要重点关注研究对象的吸烟情况。本研究在筛选SNP时，参考了Taheri等^[19]^的一项研究中筛选SNP的阈值（*P*<10^-4^），具有一定的既往研究支持。但是由于该阈值设置较高，可能会导致部分假阳性位点被纳入后续遗传变异风险的计算，从而高估遗传变异对于肺癌的影响。

在进行*Cox*回归分析时，本研究调整了遗传变异风险、性别、年龄、疾病史和生活环境评分等变量，但是由于UKB数据库中缺乏职业暴露（如职业性致癌物）等既往研究中与肺癌相关的变量信息，因此无法对这些变量进行进一步调整，可能会高估生活方式和社会经济地位对肺癌发病的影响。此外，关于生活方式和社会经济地位的信息都是参与者自报获得，可能会产生信息偏倚，并且仅采用基线收集的一次问卷，没有考虑随访期间的变化情况，后续研究可以进一步探讨生活方式和社会经济地位的动态变化对于肺癌发病的影响。本研究纳入的参与者均来自英国，这可能导致研究结果的外推性不足，且与欠发达地区相比，英国人群的社会经济地位普遍更高，会限制结果的普遍性。

本研究更为全面地揭示了生活方式和社会经济地位与肺癌发病之间的关联，以及各种生活方式与社会经济地位的交互作用，为肺癌高危人群筛查提供了科学依据，为肺癌的预防提供了新思路。
